# Lower Lid Ectropion in Hypohidrotic Ectodermal Dysplasia

**DOI:** 10.1155/2015/952834

**Published:** 2015-02-23

**Authors:** Xiaoyun Zhang, Li Xu, Xiaofang Li, Chunyan Li, Haitao Zhang

**Affiliations:** ^1^Center of Healthcare, Affiliated Hospital of Inner Mongolia Medical University, 1st North Tongdao Street, Hohhot, Inner Mongolia 010050, China; ^2^Department of Ophthalmology, Affiliated Hospital of Inner Mongolia Medical University, 1st North Tongdao Street, Hohhot, Inner Mongolia 010050, China

## Abstract

We report a case of a lower lid ectropion with ectodermal dysplasia and ectropion blepharoplasty surgery experience. A 14-year-old Han nationality male patient with typical characteristics of hypohidrotic ectodermal dysplasia presented to our clinic for his right lower lid eversion. The patient was diagnosed as having hypohidrotic ectodermal dysplasia and underwent an uneventful blepharoplasty surgery. The lower lid maintained normal position during the 10-month follow-up period. Patients with ectodermal dysplasia could firstly visit ophthalmologist for their ectropion and blepharoplasty surgery could be useful for the disease.

## 1. Introduction

The ectoderm dysplasia (ED) is a group of rare congenial inherent disorders characterized by genetic defects that result in dystrophy or agenesis of two or more tissues derived from ectodermal origin. Different combinations of affected tissues lead to that the syndrome has polymalformative clinical manifestations [1]. Most affected tissues include teeth, skin, hair, nails, and ocular tissues derived from ectoderm such as eyelid and eyelash.

The aim of this study is to report a patient with typical characteristics of hypohidrotic ED who had right lower lid ectropion secondary to dysplasia of eyelid and treated with blepharoplasty surgery. To the best of our knowledge, this is the first case reported about lower lid ectropion caused by ED and surgery experience of ectropion blepharoplasty.

## 2. Case Presentation

A 14-year-old Han nationality male patient came to our clinic for a history of 2 years of right lower lid ectropion without any significant cause, and the symptom deteriorates gradually. The patient has previously been treated with a temporary tarsorrhaphy in the same eyelid in order to solve the exposure keratitis one year ago. However, the procedure could not resolve his symptom effectively after removing the sutures one week later.

The boy was the eldest son born at term to healthy nonconsanguineous parents. No history of medication and no exposure to radiation during pregnancy were revealed. There were no other similar cases in their family including his younger sister.

**Figure 1:**
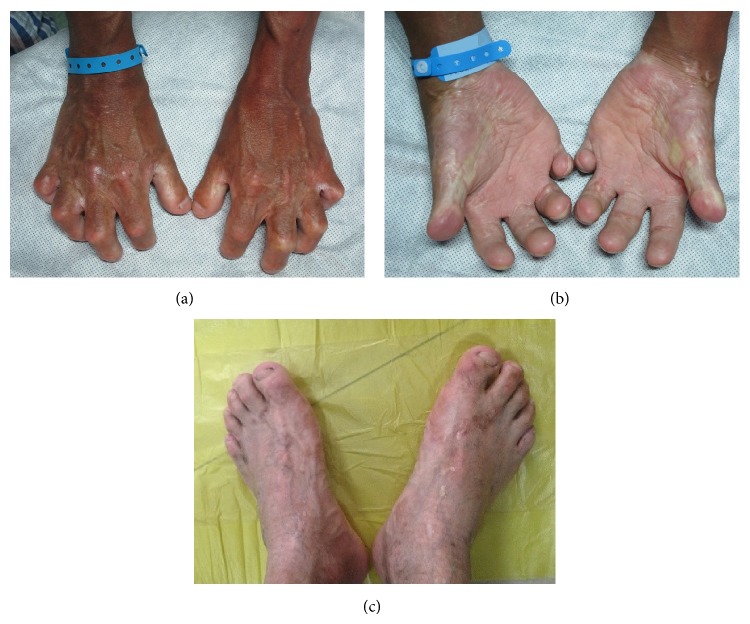
Hands and feet images. (a) Opisthenar; (b) palm; (c) feet. Clinical examination reveals fingers, especially the first and second phalange, were shorter and cannot full straighten. His palms and soles were hyperkeratosis. Fingernails and toenails were dystrophy.

The boy had normal intelligence compared with children of his age. His skin was atrophic, dry, with less sweat, with less hair, and with poor flexibility. The surface of the body skin was thin. Parents reported the boy's skin was more fragile and prone to tears and scarring. Subcutaneous blood vessels were pronounced and visible. His height was 1.89 m and weight was 70 kg. His limbs were thin and long. His low limbs were “O” type outward and lame slightly. His fingers, especially the first and second phalange, were shorter and cannot full straighten. His palms and soles were hyperkeratosis. The fingernails and toenails were dystrophy (Figures 1(a)~1(c)). Front, temporal bone and chin were prominent, while his middle face was depressed with flat nose. The proportion of his lower face was reduced.

**Figure 2:**
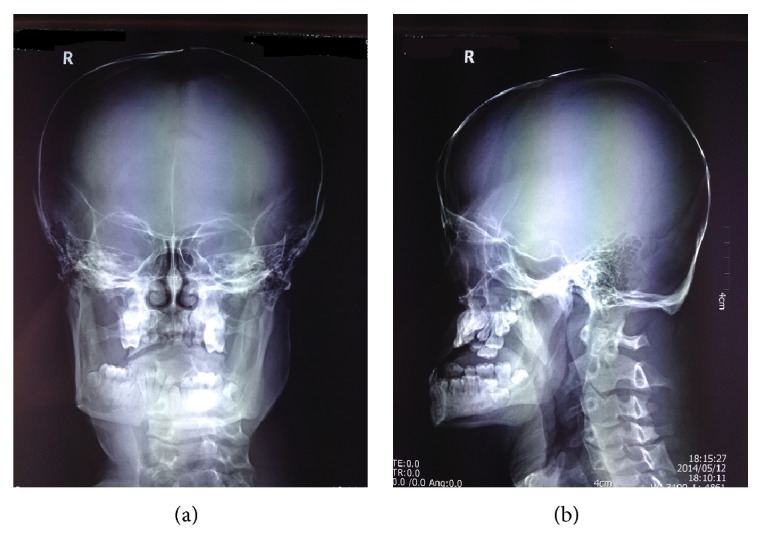
Head X-ray images. (a) Front head X-ray images presented that his teeth were sparse and irregular. Part of the permanent teeth was absent on the hypoplastic upper alveolar ridge; (b) lateral head X-ray images show that the upper alveolar ridge was depressed and lower alveolar ridge was prominent.

As his upper alveolar ridge was depressed while lower alveolar ridge was prominent, and there was wrinkle in forehead, eyelid and nasolabial groove region, leading to his aged appearance. His teeth were sparse and irregular. There was part of the permanent teeth absent on the hypoplastic upper alveolar ridge (Figures 2(a) and 2(b)).

**Figure 3:**
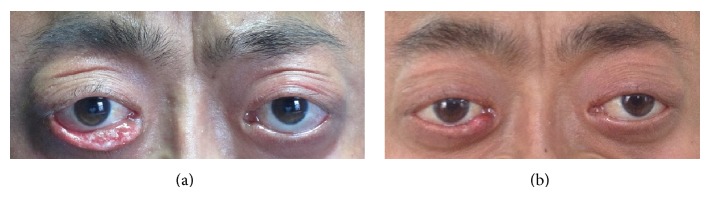
Eyes images. (a) Eyes image before surgery presented sparse eyelashes, multiple rows, or absence of lashes on the upper lids with absence of lash on the lower lids. Right lower eyelid overturned seriously; (b) eyes image at 10-month follow-up showed that right low eyelid keeps the normal position.

Examination showed that he had normal correct visual acuity and normal IOP. There were sparse eyelashes, multiple rows, or absence of lashes on the upper lids with absence of lash on the lower lids. His prominent eyes looked like golden fish eyes due to subcutaneous tissue and orbital fat atrophic. Eyelid skin was thin and loose. His right eye had hypophysis due to lower eyelid overturned seriously (Figure 3(a)). Exposed conjunctiva was dry mild conjunctival hyperemia.

On the basis of history and the presence of triad of symptoms: hypotrichosis, hypohidrosis, and hypodontia, the patient was diagnosed as having hypohidrotic ectodermal dysplasia (HED). However, multiple congenital anomalies (MCA) pattern consists of finger malformation, skin atrophic, and abnormal tall height; the patient may be a kind of new syndrome.

The patient underwent a successful ectropion blepharoplasty surgery on his right lower lid in July 18, 2013. After topical anesthesia and local injection of lidocaine with epinephrine into the portions of lower lid, the lid was split laterally into two lobes at the point of inner and outer 1/3 along the gray line for about 5 × 5 mm with a blade. The tarsus was resected about 3 × 2 mm in V shape and the corresponded lid skin was removed with a blade, respectively. After bleeding was completely stopped, the posterior and anterior lobes and the lid margin were sutured using interrupted 6-0 Vicryl, respectively, which was removed in 7 days. Postoperative eye examination showed that his right lower lid returned to normal position and no ectropion recurred during the 10-month follow-up period (Figure 3(b)).

## 3. Discussion

The ectodermal dysplasias (EDs) comprise a large, heterogeneous, diffuse, nonprogressive group of congenital inherited disorders that are defined by primary defects in the development of 2 or more tissues derived from embryonic ectoderm [1]. The tissues primarily involved are the skin and its appendages (hair follicles, eccrine glands, sebaceous glands, and nails) and teeth. Thuman first reported the disease in 1848 [2], and in 1929 it was first named as ectodermal dysplasia (ED) by Weech [3]. Based on clinical feature, Pinheiro and Freire-Maia proposed the first classification system of the ectodermal dysplasias in 1982 [4], with additional updates in 1994 and 2001 [5, 6]. There are varieties of clinical manifestation due to different combined type of affected tissues. To date, near 200 distinct disorders have been described.

We report a case of a 14-year-old Han nationality male patient with typical characteristics of hypohidrotic ED who presented with low eyelid entropion. This case had multiple tissues dysplasia, including sweat glands, skin, finger, and teeth. His skin was dry and lacks flexibility, without fine hair, subcutaneous fat layer atrophy. He had sparse and irregular teeth and some did not erupt. He also had short finger, nails, and toes deformity; his palms and soles were hyperkeratosis. The boy was abnormally tall at his age. His face looked like aged appearance. Based on clinical manifestation, we considered that the boy had a kind of congenital ectoderm dysplasia. The boy was the only case in this family and his younger sister had no such presence; it was also confirmed that it should be X-linked recessive inherent.

His ectropion was considered probably secondary to dysplasia of eyelid. The patient's lid skin was thin and subcutaneous tissue was relaxation, with lacking of flexibility, orbital fat atrophy, and the ball prominent. As affected appearance and symptom, the eyelid ectropion repair surgery should be considered. The purpose of the surgery is to return to normal anatomical position and to improve appearance. The patient underwent a successful lower lid ectropion blepharoplasty surgery to strengthen the orbicularis oculi muscle and to improve the tension of the eyelid skin to correct lid outward turning. Long-term postoperative effect and progress will be followed.
